# The Rectal Mucosal Myeloid Niche in HIV-1 Persistence: Reservoir Support, Viral Sequestration, and Therapeutic Opportunities

**DOI:** 10.3390/v18080858

**Published:** 2026-08-05

**Authors:** Hanyi Zhang, Peiming Huang, Xu Zhang, Ting Pan

**Affiliations:** 1 Key Laboratory of Tropical Disease Control of Ministry of Education, Institute of Human Virology, Department of Pathogen Biology and Biosecurity, Zhongshan School of Medicine, Sun Yat-Sen University, Guangzhou 510080, China; zhanghy558@mail2.sysu.edu.cn (H.Z.); zhxu22@mail.sysu.edu.cn (X.Z.); 2 Shenzhen Key Laboratory for Systems Medicine in Inflammatory Diseases, Zhongshan School of Medicine, Shenzhen Campus of Sun Yat-Sen University, Sun Yat-Sen University, Shenzhen 518107, China; huangpm6@mail2.sysu.edu.cn

**Keywords:** HIV-1 persistence, myeloid-associated reservoirs, rectal mucosa, myeloid cells, tissue-resident macrophages, dendritic cells, mucosal immunity, functional cure

## Abstract

Persistent HIV-1 reservoirs remain a major barrier to a durable functional cure despite long-term suppressive antiretroviral therapy. Although resting memory CD4^+^ T cells constitute the best-characterized cellular reservoir, tissue microenvironments shape viral persistence and immune clearance. The rectal mucosa represents a specialized tissue niche containing HIV-susceptible target cells, antigen-presenting cells, microbial products, inflammatory cues, and local metabolic signals. Within this setting, myeloid-lineage cells, particularly tissue-resident macrophages and dendritic cells, may contribute to HIV-1 persistence through mechanisms distinct from classical T-cell latency. Here, we review how rectal mucosal macrophages may support HIV-1 persistence through longevity, resistance to apoptosis, metabolic adaptation, epigenetic regulation, and sequestration of virions within virus-containing compartments. We also discuss the dual role of mucosal dendritic cells as sentinels that capture and transfer HIV-1 to CD4^+^ T cells, while considering the limited evidence for inducible proviral persistence in selected anatomical and cellular contexts. Importantly, we further distinguish bona fide reservoir-bearing cells from reservoir-supportive mechanisms, including viral capture, trans-infection, immune suppression, and niche-mediated protection. We also highlight how mucosal dysbiosis, barrier disruption, microbial metabolites, chronic interferon signaling, and immunoregulatory myeloid programs may stabilize HIV-1 persistence in rectal tissues. Integrating intact proviral assays, functional measurements, single-cell profiling, multiplex imaging, and spatial transcriptomics will be critical for defining myeloid-associated persistence and guiding tissue-targeted HIV-1 cure strategies.

## 1. Introduction

Achieving a functional cure for HIV-1 remains a major challenge because stable viral reservoirs persist despite effective antiretroviral therapy (ART) and immune surveillance [1,2,3]. While research has traditionally centered on the latent reservoir within resting memory CD4^+^ T cells, accumulating evidence indicates that HIV-1 persistence involves complex and multicellular processes [4,5,6]. Thus, although the CD4^+^ T-cell-centered model remains foundational, it is insufficient to fully capture the spatial and cellular heterogeneity of HIV-1 persistence in mucosal tissues.

Gut-associated lymphoid tissue (GALT), particularly the distal intestinal and rectal mucosa, represents a key anatomical site for HIV-1 persistence [4,7,8,9]. This compartment is characterized by a high frequency of CCR5^+^ and α4β7^+^ memory CD4^+^ T cells, specialized antigen-presenting cells, microbial and dietary antigen exposure, and dense lymphovascular structures [10,11,12]. Human biopsy studies further show that HIV-1 burden and immune activation patterns differ significantly between blood and gut compartments. These findings support the view that the rectal mucosa is not merely a passive reflection of the circulating reservoir, but rather represents an anatomically specialized viral niche [4,10].

However, focusing solely on the T-cell compartment provides an incomplete view of viral persistence. The rectal mucosa is a dynamic immunological niche where myeloid-lineage cells, particularly tissue-resident macrophages and dendritic cells, operate under distinct physiological and metabolic constraints [9,13,14]. Despite their significance, the contribution of these cells to HIV-1 persistence remains incompletely defined, partly because they lack the classical biomarkers used to define T-cell latency and may respond differently to current “shock-and-kill” strategies and conventional ART-based interventions [7,8].

Moreover, the unique features of the rectal niche, including chronic microbial exposure, persistent dysbiosis, epithelial barrier disruption, and distinct metabolic profiles, may further complicate the identification and targeting of myeloid-associated HIV-1 persistence. A broader framework is therefore needed to determine how the rectal microenvironment shapes myeloid and other non-T-cell contributions to viral persistence. This review aims to delineate the heterogeneous distribution and functional roles of rectal macrophages and dendritic cells, the molecular mechanisms through which these cells may support or harbor HIV-1 persistence, and the challenges they pose for HIV-1 cure efforts. By integrating insights from multi-omics and spatial biology, we propose future research directions and therapeutic strategies tailored to this tissue-specific reservoir niche, to advance the development of more comprehensive HIV-1 cure strategies.

A central unresolved issue is how to distinguish true myeloid reservoir-bearing cells from virus-associated or reservoir-supportive myeloid states, particularly in spatially complex mucosal tissues. To avoid conflating viral capture, trans-infection, and bona fide reservoir status, we first define the operational categories of HIV-1 persistence in myeloid cells used throughout this review (Table 1).

## 2. The Rectal Mucosal Niche: A Permissive Site for HIV-1 Persistence

The rectal mucosa represents a spatially compartmentalized immunological niche of the gut and has emerged as an important anatomical site for HIV-1 persistence [15]. Anatomically, this compartment is organized by a mucus-covered epithelial barrier, crypt-associated microdomains, a lamina propria rich in macrophage and dendritic cells (DCs), and dense lymphovascular drainage [9,16]. Continuous exposure to microbial and dietary antigens maintains this site in a state of tonic immune activation [14]. During HIV-1 infection, epithelial injury, microbial translocation, and persistent type I interferon signaling can amplify this state and shift it toward chronic pathological inflammation. Such activation may have divergent consequences: NF-κB, AP-1, and inflammatory cytokines may promote episodic viral transcription, whereas chronic stimulation may drive T-cell exhaustion, Th17 dysfunction, and immunoregulatory myeloid programs that reduce the clearance of infected cells [9,10,15,17,18]. Thus, mucosal inflammation should not be regarded as uniformly latency reversing or antiviral; its net effect depends on signal duration, cellular identity, and spatial context.

The microbiome and its metabolites further regulate this mucosal niche [17,19,20]. HIV infection, persistent immune activation, and ART-associated ecological shifts can reshape the gut microbial community, impair epithelial barrier integrity, and alter local metabolite availability [9,17]. These metabolites may shape the metabolic state, turnover kinetics, and functional persistence of tissue-resident macrophages and DCs [14,21]. In this context, the following sections examine how specific non-CD4^+^ cell lineages contribute to myeloid-associated persistence, viral sequestration, immune modulation, and niche-mediated HIV-1 persistence, as well as how this tissue microenvironment might be targeted in future cure strategies.

### 2.1. Macrophages in the Rectal Mucosa: Reservoir-Bearing and Reservoir-Supportive Roles

A major unresolved challenge in HIV-1 cure research is determining how tissue-resident macrophages in the rectal mucosa contribute to viral persistence. Unlike the rapid and often cytopathic viral replication observed in activated CD4^+^ T cells, macrophage-associated HIV-1 persistence may involve distinct mechanisms, including long cellular lifespan, resistance to cell death, low-level viral expression, phagocytic acquisition of infected cells, and sequestration of virions within intracellular compartments. In this sense, macrophages may function as reservoir-bearing cells in selected contexts while also contributing to reservoir-supportive components of the rectal mucosal niche.

Their anatomical positioning and cellular biology further complicate cure-directed interventions. Tissue-resident macrophages are embedded within lamina propria, perivascular, crypt-associated, and deeper intestinal microenvironments, where they are exposed to microbial products, inflammatory cytokines, hypoxia, stromal cues, and local metabolic signals. These features shield HIV-1 from immune surveillance and, in selected contexts, from optimal pharmacological pressure. Therefore, macrophage-associated persistence should be considered through a tissue-specific framework that integrates anatomical localization, viral acquisition, intracellular viral storage, immune evasion, and metabolic adaptation.

#### 2.1.1. Anatomical and Phenotypic Heterogeneity of Rectal Macrophages

Intestinal macrophage heterogeneity is closely linked to anatomical location, suggesting that HIV-1 persistence may differ across macrophage populations within the gut and rectal mucosa. These cells can be broadly divided into short-lived monocyte-derived populations and longer-lived tissue-resident populations capable of local self-maintenance. The latter are involved in vascular integrity, neuronal support, epithelial homeostasis, and tissue repair, and may provide anatomically distinct environments that differentially support HIV-1 persistence or immune evasion [9,14,22,23]. This distinction is important because rapidly replenished inflammatory monocyte-derived macrophages may contribute more to local inflammation and viral dissemination, whereas deeper, low-turnover tissue-resident macrophages may be more relevant to long-term persistence under ART.

According to anatomical location, intestinal macrophages include lamina propria macrophages, perivascular macrophages, neuron-associated macrophages, muscularis externa macrophages, and crypt- or Peyer’s patch-associated subsets [14,21,24,25,26,27]. Rather than treating these subsets as a static catalogue, their relevance to HIV-1 persistence should be understood in relation to local microenvironmental gradients. Each niche exposes macrophages to distinct levels of microbial products, oxygen tension, stromal signals, neuronal inputs, inflammatory cytokines, and lymphovascular access. These factors may shape HIV-1 entry, viral acquisition, proviral silencing, cell survival, and immune visibility [14,21,25,27].

Single-cell transcriptomic studies further support the existence of functionally diverse macrophage states in intestinal tissues, although evidence derived from non-HIV contexts, such as colorectal cancer, should be interpreted cautiously [28]. In vitro M1/M2 polarization models have shown that polarized macrophages can display altered susceptibility to HIV-1 infection, but this binary framework is insufficient to describe the complex intestinal tissue milieu [29,30]. Therefore, an anatomical and spatial classification is more informative than a simple M1/M2 dichotomy when discussing rectal macrophage-associated HIV-1 persistence.

#### 2.1.2. Routes of HIV-1 Acquisition: Infection, Phagocytosis, and Virion Sequestration

Before discussing macrophage reservoir stability, it is important to distinguish the routes by which macrophages acquire HIV-1 from the mechanisms by which they maintain viral persistence. Macrophages may become involved in HIV-1 persistence through several non-mutually exclusive pathways, including direct infection, uptake of free virions, phagocytosis of infected CD4^+^ T cells, and cell-to-cell viral transfer. These processes differ in their implications for reservoir biology: viral uptake or sequestration does not necessarily indicate the presence of an integrated, inducible provirus, whereas productive or latent infection requires stronger molecular and functional evidence. Importantly, acquisition of viral material does not necessarily indicate reservoir establishment, and evidence for bona fide reservoir status requires demonstration of integrated and inducible proviral persistence.

Direct infection of macrophages is generally less efficient than infection of activated CD4^+^ T cells because macrophages express multiple intrinsic restriction factors and maintain a cellular environment that can limit reverse transcription and nuclear import. However, once infection is established, macrophages may support low-level viral expression and long-term persistence. In addition to direct infection, macrophages can acquire HIV-1 through phagocytosis of infected T cells. Mechanistic studies have shown that HIV-1 can promote macrophage phagocytosis of infected CD4^+^ T cells by downregulating the CD47 “don’t-eat-me” signal, thereby converting phagosomes into potential sites of viral replication or viral material accumulation [31]. Macrophages can also mediate viral transfer to susceptible CD4^+^ T cells through membrane cholesterol- and DC-SIGN-dependent mechanisms, highlighting their role not only as possible reservoir-bearing cells but also as viral dissemination hubs [32,33,34].

**Figure 1:**
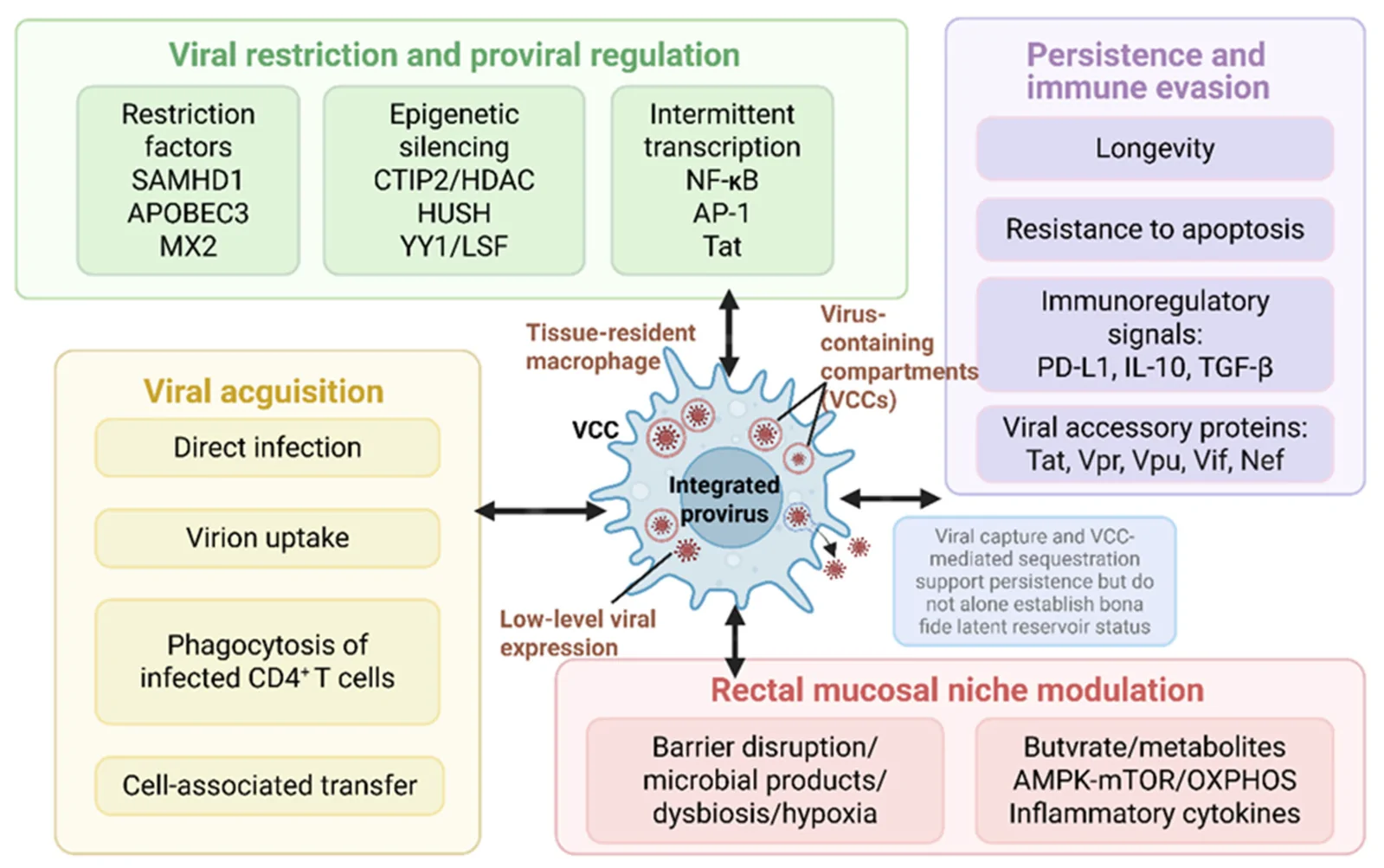
Mechanisms supporting macrophage-associated HIV-1 persistence in the rectal mucosa. Tissue-resident macrophages in the rectal mucosa may contribute to HIV-1 persistence through direct infection, virion uptake, phagocytosis of infected CD4^+^ T cells, and cell-associated viral transfer. After viral material is acquired or proviral integration occurs, persistence may be shaped by viral restriction factors, epigenetic silencing, intermittent viral transcription, long cellular lifespan, resistance to apoptosis, immunoregulatory signals, and viral accessory proteins. VCCs may sequester virions and facilitate subsequent viral transfer, but viral capture or VCC-mediated sequestration alone does not establish bona fide HIV-1 reservoir status. Local mucosal factors, including barrier disruption, microbial products, dysbiosis, hypoxia, metabolites, inflammatory cytokines, and immunometabolic pathways such as AMPK–mTOR/OXPHOS, may further regulate macrophage survival, immune evasion, and HIV-1 persistence. Created in BioRender. Zhang, H. (2026) https://BioRender.com/rbn753j.

These acquisition routes are particularly relevant in the rectal mucosa, where macrophages are positioned near epithelial surfaces, infected or activated CD4^+^ T cells, microbial products, and lymphovascular structures. As summarized in Figure 1, tissue-resident macrophages in the rectal mucosa may integrate viral acquisition, virus-containing compartment formation, proviral regulation, immune evasion, and local niche-derived signals to support HIV-1 persistence.

#### 2.1.3. Cellular Mechanisms Supporting Macrophage-Associated Persistence

Resting memory CD4^+^ T cells remain central to the stability of the HIV-1 reservoir because they can persist for long periods, undergo clonal expansion, and harbor inducible proviruses [3,35,36]. Macrophage-associated HIV-1 persistence appears to involve a different set of mechanisms. Rather than relying primarily on classical T-cell-like clonal expansion, macrophage-associated persistence may involve tissue longevity, resistance to cell death, low-level viral expression, phagocytic viral acquisition, and protected intracellular virion storage [13,26,37].

Tissue-resident macrophages represent favorable cellular niches for viral persistence because of their long lifespan and low turnover. Unlike circulating monocytes, which are rapidly replenished, tissue macrophages can maintain local population homeostasis through self-renewal and long-term residence [14,21]. Under inflammatory conditions, these cells may expand locally and persist independently of the peripheral CD4^+^ T-cell pool, potentially contributing to tissue-based viral persistence [14,23]. Animal models provide additional support for a macrophage contribution to persistence and rebound: after ART interruption, bone marrow macrophages can contribute to viral rebound, and under conditions of CD4^+^ T-cell depletion, macrophages may become a dominant cell type supporting persistent lentiviral replication [38,39]. However, these findings should be interpreted as supportive rather than definitive evidence for rectal macrophage reservoirs in all clinical contexts.

Macrophages also exhibit marked resistance to HIV-1-induced cytopathic effects, which may favor reservoir stability. Compared with activated CD4^+^ T cells, infected macrophages can survive for prolonged periods and maintain viral material without rapid cell death. This anti-apoptotic phenotype may allow infected or virus-bearing macrophages to persist in tissues despite ART and immune pressure. In intestinal tissues from individuals receiving long-term ART, macrophages carrying intact proviruses have been detected, supporting the possibility that macrophages can contribute to persistent tissue reservoirs in vivo [40].

A distinctive feature of macrophage-associated HIV-1 persistence is the formation of virus-containing compartments (VCCs). Unlike the predominant assembly and budding of viral particles at the plasma membrane in CD4^+^ T cells, HIV-1 can accumulate within intracellular, plasma membrane-connected or late endosome-like compartments in macrophages [13,41,42]. These compartments may sequester viral particles from direct extracellular immune recognition, preserve infectious virions, and facilitate subsequent cell-to-cell transfer to susceptible CD4^+^ T cells. Importantly, VCCs provide a macrophage-specific layer of physical protection that differs from classical proviral latency. The presence of VCCs does not by itself establish a bona fide viral reservoir; rather, VCCs should be viewed as a mechanism of virion sequestration and reservoir support unless accompanied by demonstration of replication-competent provirus.

#### 2.1.4. Restriction Factors, Proviral Regulation, and Immune Evasion

Macrophage-associated HIV-1 persistence reflects a balance between restriction-factor-mediated resistance to infection and post-integration mechanisms that suppress, sustain, or intermittently reactivate proviral transcription. Before integration, host restriction factors form a first line of defense. SAMHD1 blocks viral DNA synthesis by depleting intracellular dNTP pools [43,44,45], APOBEC3 family proteins induce cytidine deamination and viral genome editing [43,46], and MX2 interferes with nuclear import of pre-integration complexes [47]. These mechanisms help explain why productive infection of macrophages is often inefficient.

Once integration occurs, the regulatory focus shifts from blocking infection to controlling proviral transcription. HIV-1 preferentially integrates into transcriptionally active genomic regions under the guidance of LEDGF/p75 [43], but integration does not necessarily result in efficient viral gene expression. Reversible epigenetic reprogramming can establish transcriptional silencing through chromatin condensation and recruitment of repressive complexes. In macrophages, CTIP2-centered repressive complexes can recruit epigenetic modifiers such as HDACs to the viral promoter, while host transcriptional repressors such as YY1 and LSF may cooperate with HDAC-containing complexes to suppress HIV-1 LTR activity [40,48,49]. HUSH-associated heterochromatin mechanisms may also contribute to position-dependent proviral silencing, particularly when proviruses reside in less transcriptionally permissive genomic regions [50]. In individuals on long-term ART, intact proviruses can be enriched in non-genic and heterochromatic regions, providing a structural basis for deep transcriptional silencing [51].

Post-transcriptional regulation may further limit viral gene expression. Specific miRNAs, including miR-28 and miR-150, can promote viral mRNA degradation, whereas the RNA-binding protein G3BP1 can mediate translational inhibition [43]. TREX1 clears exposed cytoplasmic viral DNA to evade innate immune sensing, and MATR3 regulates viral protein production by controlling nucleocytoplasmic transport of viral mRNA [43]. Together, these mechanisms may allow infected macrophages to maintain a low-transcription or intermittently active state compatible with long-term persistence.

HIV-1 accessory proteins also remodel macrophage antiviral responses. That promotes viral transcription while modulating macrophage immune functions [22,43]. Vpu promotes viral release and counteracts BST-2/tetherin, while also contributing to immune evasion through effects on host restriction and recognition pathways [23,31,52]. Vpr can weaken antiviral responses by inhibiting IFITM3 and IRF3 while sustaining IL-6 expression [23,48,53], and Vif targets APOBEC3 proteins for degradation, enabling more efficient replication in macrophages [46,52]. Unlike HIV-2 or several SIV lineages, HIV-1 does not encode Vpx; therefore, direct SAMHD1 degradation by Vpx should be discussed only as a comparative lentiviral mechanism rather than an HIV-1 mechanism. Persistent virus-driven inflammatory signaling may further reprogram macrophages through pathways such as NF-κB, upregulating PD-L1 and inducing TGF-β and IL-10 secretion, thereby creating an immunoregulatory local milieu that reduces clearance of infected cells [23].

Although clonal expansion is a central mechanism of reservoir maintenance in CD4^+^ T cells, its role in macrophages appears more limited and mechanistically distinct. In CD4^+^ T cells, clonal expansion can substantially contribute to reservoir persistence and ongoing virus production under ART [35,36,54]. In contrast, macrophage-associated persistence appears to rely more heavily on intrinsic cellular properties, including longevity, resistance to apoptosis, low-level viral replication, and tissue retention. Integration-site studies suggest that macrophages may display a more restricted repertoire of accessible host genes than CD4^+^ T cells [37], and experimental studies indicate that HIV-1 infection or Tat expression may promote macrophage survival, migration, phagocytosis, or proliferation under selected conditions [22]. Identical viral clones detected across different tissues may reflect inter-tissue migration or shared viral sources, but this should not be interpreted as definitive evidence that macrophage clonal expansion is a dominant mechanism of reservoir maintenance [55]. Thus, classical clonal expansion is better established in CD4^+^ T-cell reservoirs, whereas macrophage-associated persistence is more plausibly driven by longevity, survival, viral sequestration, and tissue-specific immune evasion.

#### 2.1.5. Immunometabolic Regulation of Macrophage-Associated Persistence

Metabolic adaptation is increasingly recognized as a factor influencing macrophage survival, inflammatory function, and susceptibility to HIV-1 persistence. Macrophages can maintain long-lived and relatively quiescent states through metabolic programs partly dependent on oxidative phosphorylation (OXPHOS), which may support survival under ART and inflammatory pressure [56]. In the rectal mucosa, local hypoxia, microbial metabolites, epithelial injury, and inflammatory cytokines may reshape tissue-resident macrophage metabolism, thereby influencing viral persistence and immune function [21,25].

Several metabolic pathways may be relevant to macrophage-associated HIV-1 persistence. OXPHOS can maintain mitochondrial membrane potential and limit excessive reactive oxygen species production, supporting macrophage survival. The AMPK–mTOR axis regulates the balance between cellular metabolism, activation state, and viral latency [49,56]. Local metabolites such as butyrate may influence chromatin accessibility, inflammatory polarization, and survival programs, although their effects are likely context dependent rather than uniformly proviral or antiviral. ATRA may exert bidirectional effects by enhancing macrophage susceptibility to HIV-1 through mTOR activation while also upregulating ABCA1, which can disrupt lipid raft structures important for viral entry [49,57]. These examples illustrate how the same metabolic or differentiation signal may have opposing effects depending on cell state, tissue context, and timing.

Metabolic regulation also intersects with innate immune sensing. The MDA5–MAVS–IRF5 axis can recognize HIV-1 intron-containing RNA and induce inflammatory responses, with its activation coupled to OXPHOS status [58]. In aged macrophages, increased GLUT1-mediated glycolysis may contribute to inflammatory activation and immunosenescence, suggesting that metabolic dysregulation could affect HIV-associated tissue inflammation and reservoir persistence [59]. Metabolic modulators such as metformin or rapamycin may therefore be useful experimental tools for probing macrophage immunometabolism, although their therapeutic relevance for HIV cure requires careful validation. Overall, OXPHOS, AMPK–mTOR signaling, innate sensing pathways, ATRA-related programs, and microbial metabolites represent potentially modifiable axes for regulating macrophage-associated persistence.

#### 2.1.6. Translational Implications and Unresolved Questions

Targeting macrophage-associated HIV-1 persistence raises several macrophage-specific translational challenges. First, detection remains difficult. Tissue-resident macrophages are scarce, spatially heterogeneous, and difficult to isolate from small rectal biopsy samples. Enzymatic digestion can alter transcriptional states or lead to loss of fragile virus-bearing cells. Conventional bulk PCR cannot determine whether proviral DNA originates from T cells, macrophages, dendritic cells, or other neighboring populations, and it cannot reliably distinguish intact from defective proviruses without additional assays [11,15,32,37,60]. Therefore, identifying macrophage-associated HIV-1 persistence requires approaches that combine cell-type resolution, intact proviral measurements, functional inducibility assays, and spatial validation.

Second, therapeutic delivery to rectal tissue macrophages remains uncertain. ART penetration and pharmacodynamic activity may vary across tissue compartments, and tissue-resident macrophages may reside in microenvironments with limited drug access, altered pH, hypoxia, or dense extracellular matrix structures [9]. Moreover, CD4^+^ T-cell-oriented latency-reversing agents may not efficiently target macrophage-associated HIV-1 persistence because macrophage infection is regulated by distinct restriction factors, epigenetic programs, innate immune pathways, and metabolic states [43,45,61].

Third, macrophage clearance strategies must balance efficacy and safety. Macrophages are essential for mucosal homeostasis, microbial containment, epithelial repair, and tissue remodeling. Broad depletion or excessive activation of macrophages could therefore worsen barrier dysfunction and inflammation. Emerging approaches include myeloid-tailored latency modulation, inhibition of pro-survival pathways, capsid- or host factor-targeted strategies, activation of inflammasome pathways such as CARD8, and interventions targeting viral accessory proteins or host factors such as LAPTM5 [2,43,48,62,63,64]. Additional strategies, including modulation of macrophage polarization, anti-inflammatory interventions such as anti-TNF therapy, and metabolic reprogramming, may help remodel the local niche [21,23,26]. However, these approaches should be viewed as investigational and will require validation in tissue-relevant models and clinical samples.

Together, these considerations indicate that macrophage-directed cure strategies should not simply mimic CD4^+^ T-cell-centered “shock-and-kill” approaches. Instead, they require a combination of tissue-level detection, myeloid-specific latency modulation, improved drug delivery, controlled immune clearance, and preservation of essential mucosal macrophage functions.

### 2.2. Dendritic Cells in the Rectal Mucosa: Viral Capture, Immune Regulation, and Reservoir-Supportive Roles

While macrophages may contribute to HIV-1 persistence through longevity, intracellular virion sequestration, and tissue residency, dendritic cells occupy a unique position between mucosal barrier sensing and adaptive immunity. DCs coordinate immune surveillance, antigen presentation, and T-cell priming at mucosal surfaces [8,65]. In the genital, rectal, and anogenital mucosa, they are often among the first innate immune cells to encounter HIV-1 and can influence early viral dissemination, local immune activation, and early reservoir seeding [8,65,66,67,68]. Their contribution to HIV-1 persistence should therefore be understood not only in terms of direct infection, but also through their capacity to capture virions, transfer virus to CD4^+^ T cells, regulate interferon and inflammatory responses, and shape the tissue microenvironment.

DCs can capture HIV-1 through CD4, DC-SIGN, mannose receptors, C-type lectin receptors, and related pathways, and subsequently promote viral dissemination through antigen presentation, infectious synapse formation, and migration to draining lymphoid tissues [69,70]. However, viral capture and transfer should not be equated with bona fide latent infection. In most settings, DCs are less susceptible to productive HIV-1 infection than activated CD4^+^ T cells, and the evidence for long-term inducible proviral persistence is limited to selected DC subsets and anatomical contexts. Therefore, rectal mucosal DCs are best considered primarily as reservoir-supportive components of the HIV-1 persistence niche, with selected populations potentially exhibiting reservoir-bearing characteristics under defined anatomical and cellular contexts.

#### 2.2.1. Subset Specialization and Rectal/Anogenital Relevance

Modern DC classification recognizes conventional DC1 (cDC1), conventional DC2 (cDC2), plasmacytoid DCs (pDCs), and monocyte-derived DCs (moDCs) that arise under inflammatory conditions [66,67]. For understanding rectal and anogenital HIV-1 persistence, the classification should focus on subsets with direct relevance to mucosal viral encounter, CD4^+^ T-cell interaction, and immune regulation. These include Langerin^+^ cDC2, CD14^+^ CD1c^+^ moDCs, and pDCs, while cDC1, classical Langerhans cells, and follicular dendritic cells may be discussed as functional comparators.

Langerin^+^ cDC2 cells have recently been identified as a distinct DC subset enriched in anorectal mucosa, with functional and phenotypic features that differ from epidermal Langerhans cells [71]. These cells express CD1c, CD11c, DC-SIGN, HLA-DR, and Langerin, combining cDC2-like antigen-presenting capacity with molecules involved in viral binding and capture [66,69,71,72]. Because Langerin^+^ cDC2 cells are enriched at mucosal exposure sites and can interact closely with CD4^+^ T cells, they are particularly relevant to early HIV-1 capture and potential local viral transfer in the rectal/anogenital mucosa. Functionally, Langerin^+^ cDC2 cells can activate CD4^+^ T cells through HLA class II-mediated antigen presentation, but this same DC–T-cell interaction may also create opportunities for HIV-1 transfer to susceptible CD4^+^ T cells [66,67,71]. Thus, their role is context dependent: they may contribute to antiviral immune coordination while simultaneously facilitating viral dissemination under permissive conditions.

pDCs play a different role in the mucosal HIV-1 niche. They recognize viral nucleic acids through TLR7/9 signaling and rapidly produce type I interferons, thereby contributing to early antiviral defense [66,67,73]. Stronger pDC-associated interferon responses have been associated with smaller measured reservoir sizes in some clinical contexts [73]. However, chronic or dysregulated type I interferon signaling may also promote immune exhaustion, myeloid activation, tissue dysfunction, and impaired clearance of infected cells. Therefore, pDCs should be framed primarily as regulators of the reservoir niche rather than as major rectal reservoir-bearing cells.

Other DC-related populations contribute to antiviral immunity but are less central to the rectal myeloid-persistence argument. cDC1 specialize in cross-presentation and CD8^+^ T-cell activation, and their restriction-factor profile may limit productive infection [66,74,75]. Classical Langerhans cells can capture and degrade HIV-1 through Langerin under some conditions, although high viral exposure or alternative entry pathways may permit viral transfer [69,72,76]. Follicular dendritic cells can retain immune-complexed virions in lymphoid follicles without being productively infected, thereby supporting viral persistence through extracellular virion retention rather than classical cellular infection [77]. These populations provide important functional comparators, but the discussion of rectal and anogenital tissues should remain centered on Langerin^+^ cDC2 cells and inflammatory moDCs as HIV-interacting and transfer-relevant populations.

#### 2.2.2. Viral Capture and Transfer: Reservoir-Supportive Mechanisms

DCs play a central role in mucosal HIV-1 transmission by capturing virions at barrier sites and transferring them to susceptible CD4^+^ T cells. During trans-infection, DCs bind and internalize HIV-1 through C-type lectin receptors, DC-SIGN-related pathways, actin-rich dendrites, and non-degradative endocytic compartments. They may then transfer intact virions to CD4^+^ T cells through infectious synapses [8,78,79]. This process allows DCs to amplify local infection without necessarily being productively infected themselves.

Different DC subsets may support viral transfer through distinct mechanisms. Langerin^+^ cDC2 cells may use DC-SIGN- and Siglec-1/CD169-associated pathways to retain virions and facilitate transfer within rectal tissues and draining lymphoid structures [71,80]. cDC2 and moDCs are closely linked to CD4^+^ T-cell priming and may therefore provide efficient cellular contacts for HIV-1 transmission in mucosal tissues [74]. pDCs can mediate cell-contact-dependent viral transfer while also shaping the antiviral environment through type I interferon production [81,82]. These functions position DCs as important reservoir-supportive components because they can promote viral spread, enhance local CD4^+^ T-cell infection, and shape immune activation. However, these transmission-related functions should be distinguished from direct evidence of an integrated, inducible, replication-competent reservoir.

In addition to trans-infection, DCs may support cis-infection under selected conditions, although productive replication is generally inefficient compared with activated CD4^+^ T cells. Cis-infection requires that HIV-1 overcome intrinsic restriction pathways and establish viral gene expression within DCs. Therefore, the classification of DCs as bona fide HIV-1 reservoir-bearing cells should be applied cautiously and requires evidence of integrated proviral DNA, inducible viral RNA or protein expression, and, when possible, replication-competent virus recovery.

**Figure 2:**
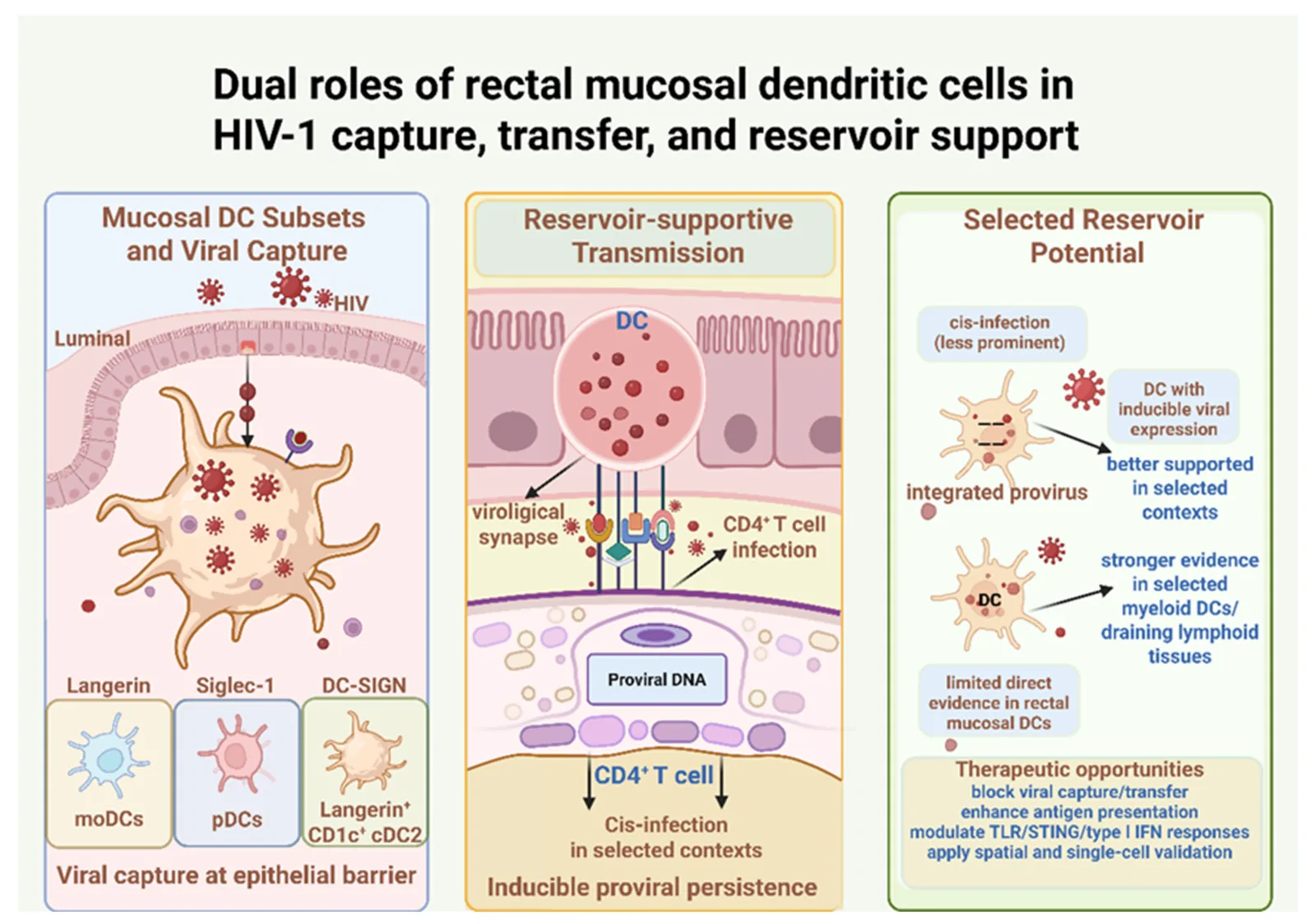
Dendritic cell-associated mechanisms in rectal mucosal HIV-1 persistence. Rectal mucosal dendritic cell subsets, including Langerin^+^ CD1c^+^ cDC2 cells, moDCs, and pDCs, can capture HIV-1 at epithelial barrier sites through viral-binding receptors such as DC-SIGN, Langerin, and Siglec-1. Virus-associated DCs may retain virions and transfer them to susceptible CD4^+^ T cells through virological synapses, thereby promoting local dissemination, antigen presentation, and type I interferon/inflammatory signaling. These cells may also shape antigen presentation and local T-cell activation. In selected anatomical and cellular contexts, some myeloid DC populations may support and inducible proviral persistence. However, viral capture and trans-infection should not be equated with bona fide viral reservoir status. Potential therapeutic and validation strategies include blocking viral capture or transfer, enhancing antigen presentation, modulating TLR/STING/type I interferon responses, and applying spatial and single-cell approaches to distinguish reservoir-bearing DCs from reservoir-supportive DCs. Created in BioRender. Zhang, H. (2026) https://BioRender.com/bo18k6c.

Together, these findings indicate that rectal mucosal DCs contribute to HIV-1 persistence primarily through viral capture, retention, and transfer, whereas their capacity to function as bona fide reservoir-bearing cells requires stricter evidence of integrated and inducible provirus. As summarized in Figure 2, DC-associated HIV-1 persistence spans reservoir-supportive viral capture and transfer, selected-context cis-infection, and inducible proviral persistence.

#### 2.2.3. Evidence for DC-Associated Reservoir Potential

Current evidence does not support a broad classification of rectal mucosal DCs as major HIV reservoirs. However, this does not exclude the possibility that selected DC populations in specific anatomical and cellular contexts may fulfill reservoir criteria. Therefore, DC-associated persistence should be interpreted as a spectrum, ranging from reservoir-supportive functions, including viral capture and trans-infection to selected-context integrated and inducible proviral persistence. Although DCs are generally less permissive to productive HIV-1 infection than activated CD4^+^ T cells, selected myeloid DC populations may harbor persistent proviral genomes under defined anatomical and clinical conditions [40,68]. Viral capture, extracellular retention, or non-degradative storage of virions does not by itself prove bona fide reservoir status. Stronger evidence requires integrated proviral genomes together with inducible viral expression or recovery of replication-competent virus. Productive infection alone indicates active viral replication but does not necessarily establish a latent reservoir unless integrated and inducible proviral persistence is demonstrated.

Several mechanisms may support DC-associated persistence or reservoir-supportive functions. Virus-associated or potentially infected DCs may exhibit altered antiviral, inflammatory, and metabolic programs that favor viral persistence [68]. Persistent antigen exposure and interferon signaling can induce PD-L1 expression, impair T-cell function, and promote inflammatory cytokine production through DC-associated reverse signaling pathways [83]. Persistence-associated phenotypes may also involve precursor proliferation, differentiation, or anti-apoptotic programs under selected conditions [55]. TLR7/8 agonists can directly induce viral RNA and protein production in DCs without requiring CD4^+^ T cells, supporting the possibility that some DC populations may function as inducible viral sources in defined contexts [84]. Moreover, interactions between HIV-1 and DCs may influence viral adaptation, transmission efficiency, and pathogenicity [67].

Recent evidence indicates that myeloid DC subsets in mesenteric lymph nodes draining the rectal region can harbor intact, inducible, replication-competent HIV-1 proviruses during long-term suppressive ART [55,84]. These findings suggest that DCs can meet reservoir criteria in specific lymphoid contexts, although direct evidence for bona fide latent reservoirs within rectal mucosal DCs remains more limited. Thus DC-associated persistence should be evaluated according to anatomical location, cellular identity, and functional evidence rather than by a binary reservoir/non-reservoir classification. Similar evidence has also been reported in other myeloid populations, including tissue macrophages and microglia, where persistent HIV DNA and, in selected contexts, inducible replication-competent virus have been detected during suppressive ART [5,85,86]. These findings further support the concept that myeloid-associated HIV persistence represents a tissue- and cell-type-dependent phenomenon rather than a DC-specific process. Together, these findings support a cautious but important conclusion: rectal and draining-lymphoid DCs are unlikely to function only as passive virion carriers, but may contribute to HIV-1 persistence through viral dissemination, immune modulation, and, in selected contexts, inducible proviral persistence. Their role in HIV-1 cure strategies should therefore be evaluated with assays that distinguish viral capture, evidence of productive infection, and bona fide reservoir-bearing states.

#### 2.2.4. Therapeutic Implications and Unresolved Questions

DC-targeted strategies for HIV-1 cure may need to address two related goals: limiting DC-mediated viral transfer and enhancing DC-dependent immune clearance. One approach is to strengthen the immune-activating functions of DCs using TLR agonists, cGAS–STING pathway activators, or other immunomodulators that enhance antigen presentation and improve virus-specific T-cell responses [75,87]. Another approach is to inhibit DC-mediated viral capture and transfer by targeting viral binding and trafficking pathways, including DC-SIGN, Siglec-1/CD169-associated mechanisms, TSPAN7, DNM2, or macropinocytosis-related pathways [79,80,88]. These strategies may reduce the reservoir-supportive role of DCs by limiting cell-to-cell viral dissemination.

Combination immunomodulatory strategies may also be necessary. PD-1/PD-L1 pathway blockade may improve exhausted T-cell function in selected contexts, while modulation of type I interferon signaling could help restore a more balanced antiviral environment [1,63,83,89]. CCR5 blockade may reduce new rounds of infection while DC activation exposes viral antigens or improves immune priming. However, these approaches must be carefully calibrated because excessive DC activation could worsen inflammation, expand susceptible CD4^+^ T-cell targets, or reinforce immune exhaustion.

Several unresolved challenges remain. First, most current latency-reversing and immune-clearance strategies have been optimized for CD4^+^ T-cell reservoirs rather than DC-associated persistence. Quiescent or tissue-resident DC populations may respond differently to LRAs, innate immune agonists, or checkpoint blockade [1,68,77]. Second, DC heterogeneity complicates therapeutic targeting: interventions that activate one subset may suppress or dysregulate another. Third, definitive markers of DC-associated reservoirs are lacking, and bulk assays cannot reliably distinguish virus captured by DCs from integrated proviruses within DCs. Fourth, DCs are essential for mucosal immune surveillance, tolerance, and tissue homeostasis, so broad depletion or uncontrolled activation may be harmful.

Future work should prioritize DC subset-specific detection and intervention strategies. This includes combining sorted or spatially resolved DC profiling with intact proviral measurements, inducibility assays, single-cell multi-omics, and multiplex imaging. Therapeutically, DC-targeted delivery systems, miRNA-based regulation of latency-related pathways such as the miR-17/92 cluster, and subset-selective innate immune modulation may offer opportunities to manipulate DC-associated HIV-1 persistence while preserving normal immune function [40]. Current evidence suggests that rectal mucosal and draining-lymphoid DCs contribute to HIV-1 persistence primarily through viral capture, dissemination, and immune regulation, while only selected subsets may harbor inducible proviruses. Therefore, therapeutic strategies should selectively target pathogenic DC-associated functions while preserving their essential roles in mucosal immune regulation [75].

## 3. Challenges in Detecting and Quantifying Rectal Myeloid-Associated HIV-1 Persistence

Detecting myeloid-associated HIV-1 persistence in rectal tissue is difficult because conventional assays cannot readily distinguish cell-intrinsic proviral infection from captured virions, engulfed infected cells, or neighboring T-cell-derived signals. A comprehensive analytical framework should therefore address four key dimensions: cellular identity, proviral integration and intactness, inducibility or replication competence, and spatial localization.

### 3.1. Limitations of Conventional Assays in Tissue Compartments

Standard methodologies for measuring the HIV-1 reservoir, including the quantitative viral outgrowth assay (QVOA) and bulk PCR-based quantification of HIV DNA, have important limitations when applied to rectal tissue compartments. QVOA was originally optimized for resting memory CD4^+^ T cells and may underestimate myeloid-associated persistence, which can differ in activation requirements, viral transcriptional control, and susceptibility to ex vivo stimulation. Moreover, QVOA detects only inducible replication-competent virus under the specific culture conditions used, and therefore may fail to capture low-frequency or tissue-specific myeloid-associated viral persistence.

Bulk PCR-based assays provide higher sensitivity for detecting HIV DNA but lack cellular and spatial resolution. Total HIV-1 DNA measurements cannot distinguish intact from defective proviruses, and even intact proviral assays cannot by themselves determine whether the signal originates from CD4^+^ T cells, macrophages, dendritic cells, or other neighboring populations. This limitation is particularly important in rectal biopsies, where multiple immune-cell subsets coexist in close proximity. Bulk approaches may therefore mask the relative contribution of non-CD4^+^ cell populations to tissue-associated HIV-1 persistence.

Technical processing of rectal biopsies introduces additional challenges. Enzymatic digestion, mechanical dissociation, and cell sorting can alter transcriptional profiles, disrupt cell–cell interactions, and result in the loss of fragile or rare virus-associated myeloid subsets. These procedures may also obscure the original anatomical localization of HIV-associated signals and their relationship with surrounding cellular niches. As a result, ex vivo analyses may not fully represent the in situ distribution, functional state, or microenvironmental context of HIV-1 persistence within the rectal mucosa.

### 3.2. Advances in in Situ Imaging and Single-Cell Spatial Technologies

High-resolution spatial approaches have improved the study of mucosal HIV-1 persistence by enabling viral signals to be interpreted within preserved or partially preserved tissue architecture. Single-cell transcriptomic and multi-omic approaches enable detailed characterization of cellular phenotypes, activation states, restriction-factor expression, and transcriptional programs within infected or virus-associated tissues [90]. However, dissociation-based methods alone lose spatial context and cannot define the anatomical relationships between infected cells and their surrounding microenvironment. Moreover, current spatial approaches remain subject to technical limitations, including tissue fixation-associated loss of RNA integrity, imperfect cell segmentation, and the need for rigorous negative controls to distinguish true HIV-1 signals from background noise [91]. Accordingly, spatial transcriptomics should be integrated with multiplex immunofluorescence, RNAscope or DNAscope-based viral detection, and in situ protein validation to accurately localize viral genomes or transcripts within the tissue microenvironment.

Recent advances have enabled the identification of candidate reservoir-associated signatures using spatial transcriptomics, as well as integrated single-cell approaches that reveal epigenetic repressors, inflammatory pathways, and autophagy-related programs in treatment-resistant myeloid cells [92]. These technologies are particularly relevant for rectal mucosal studies because they can help assess whether HIV-associated signals localize near epithelial barriers, crypt-associated regions, lymphovascular structures, macrophage-rich niches, or DC–T-cell interaction zones. Such spatial information is valuable for distinguishing direct cellular infection from virion capture, trans-infection, and reservoir-supportive tissue niche.

Despite these advances, spatial approaches remain technically challenging in rectal mucosal tissues. Tissue fixation and processing may affect RNA preservation and antigen accessibility, while antibody specificity and probe efficiency can influence signal interpretation. In addition, limited tissue availability and the complex cellular composition of mucosal biopsies may constrain sensitivity and quantitative assessment. Therefore, spatial technologies should be integrated with complementary molecular and functional assays rather than interpreted as standalone approaches.

A stepwise validation workflow should therefore combine tissue-level localization with cell-type-specific molecular and functional analyses. These findings require orthogonal validation because technical limitations may influence signal interpretation. Intact proviral DNA assays, near-full-length sequencing, integration-site analyses, viral RNA induction assays, and viral outgrowth approaches provide complementary evidence to distinguish intact and inducible reservoirs from defective or non-productive viral signals. Combined with cell sorting or spatial detection, these approaches can better define whether rectal macrophages or dendritic cells harbor bona fide viral reservoirs or primarily support HIV-1 persistence through viral retention, trans-infection and immune modulation.

Immuno-PET imaging may offer a complementary strategy for whole-body visualization of viral reservoirs and tissue-level viral persistence, although its cellular resolution remains limited [93]. Therefore, future detection strategies should not rely on any single platform. Instead, a robust validation framework should integrate intact proviral measurements, functional inducibility assays, single-cell profiling, multiplex imaging, and spatial validation. This integrated approach will be important for resolving the complexity of tissue-associated HIV-1 persistence and for determining which myeloid populations represent true reservoir-bearing cells, reservoir-supportive niches, or both. Evidence for myeloid-associated HIV-1 persistence should be interpreted along a continuum, ranging from viral capture and reservoir-supportive functions to bona fide reservoir-bearing states supported by integrated and inducible proviral evidence.

## 4. Future Therapeutic Strategies: A Multi-Cellular Translational Roadmap

Because rectal myeloid-associated HIV-1 persistence reflects both cell-intrinsic mechanisms and tissue-level constraints, therapeutic strategies should target not only reservoir-bearing or virus-associated cells, but also the mucosal environments that sustain them. The following sections outline three complementary directions: combination eradication approaches, tissue-targeted drug delivery, and microenvironmental or immunometabolic remodeling.

### 4.1. From Monotherapy to Combinatorial Synergy

Current HIV-1 cure strategies increasingly recognize that no single intervention is likely to eliminate tissue-associated reservoirs across diverse cellular and anatomical compartments. The conventional “shock-and-kill” strategy aims to reverse latency and expose infected cells to immune clearance, but it faces important limitations in tissue-resident myeloid cells. Latency-reversing agents (LRAs) may activate HIV-1 transcription while simultaneously inducing anti-apoptotic, inflammatory, or autophagy-related survival programs that protect infected cells from elimination [94]. This concern is particularly relevant for macrophages and dendritic cells, whose survival, activation state, and antiviral functions are tightly regulated by local tissue cues.

These limitations support the use of combination approaches. The SECH strategy, which combines latency reversal with inhibition of pro-survival pathways, has shown activity against HIV-1 in patient-derived macrophages and has been associated with rebound-free viral control in humanized mouse models [95,96]. Immune-enhancing strategies may further improve clearance. For example, the IL-15 superagonist N-803 can enhance NK-cell and T-cell effector functions and may synergize with other interventions to reduce reservoir burden [97,98]. In parallel, “block-and-lock” approaches seek to reinforce durable transcriptional silencing of proviruses through epigenetic regulation, providing an alternative to latency reversal when complete clearance is not feasible [99]. These examples underscore the importance of tailoring HIV cure approaches to both the cellular identity of reservoir-associated cells and the tissue microenvironment in which they persist.

### 4.2. Microenvironment-Targeted Drug Delivery and Pharmacological Strategies

Targeting rectal myeloid-associated HIV-1 persistence requires overcoming anatomical and pharmacological barriers within the mucosal tissue. The mucus layer, epithelial tight junctions, extracellular matrix, local inflammation, and lymphovascular architecture can all influence drug penetration, retention, and exposure at the relevant cellular sites [9]. Therefore, reservoir-directed therapy should consider not only systemic drug potency, but also local pharmacokinetics and pharmacodynamics within the rectal mucosa.

Nanoparticle-based formulations, including PLGA particles and liposomes, may improve mucosal retention and cell-specific delivery [100]. CD169-directed, HIV-mimicking antiretroviral nanoparticles provide another example of a myeloid-targeted strategy, with the potential to enable selective delivery to lymph node-resident myeloid cells and prolonged drug retention within tissue-associated persistence sites [5,101]. Prodrug systems activated by macrophage-enriched enzymes may further support tissue-restricted pharmacology and reduce systemic toxicity [102]. These approaches are particularly relevant for macrophages and dendritic cells because their anatomical localization, endocytic capacity, and tissue residency may be exploited for selective drug delivery.

Mechanism-based pharmacokinetic–pharmacodynamic modeling can provide a quantitative framework for optimizing these interventions. By integrating formulation-dependent exposure, target-site drug persistence, cellular uptake, antiviral activity, and latency-modulating effects, PK–PD modeling may help guide rational dose selection and regimen design for reservoir-directed therapies [103,104]. Future studies should therefore pair tissue-targeted delivery platforms with direct measurements of drug distribution, myeloid-cell exposure, and reservoir activity in rectal mucosal samples.

### 4.3. Microenvironmental and Immunometabolic Engineering

The rectal mucosal reservoir niche is shaped not only by infected cells themselves, but also by hypoxia, microbial metabolites, epithelial barrier integrity, inflammatory cytokines, and tissue-repair signals. Treatment-resistant myeloid cells may upregulate glycolytic, autophagy-related, and anti-apoptotic programs, suggesting that metabolic adaptation contributes to therapeutic resistance [95]. Therefore, immunometabolic engineering should be considered as a complementary strategy to conventional latency reversal, immune checkpoint modulation, or cytotoxic clearance.

Microbial metabolites may influence HIV-1 latency and the survival of reservoir-associated cells by modulating histone deacetylase activity, chromatin accessibility, macrophage polarization, dendritic-cell activation, and T-cell differentiation [19]. However, these effects are context dependent and should not be reduced to a simple pro-viral or antiviral model. For example, the same metabolite may suppress inflammation in one setting but alter chromatin accessibility or immune activation in another. Future strategies may involve restoring epithelial barrier integrity, reducing pathological inflammation, modulating microbial metabolite profiles, and selectively targeting survival pathways in infected or virus-associated myeloid cells.

**Figure 3:**
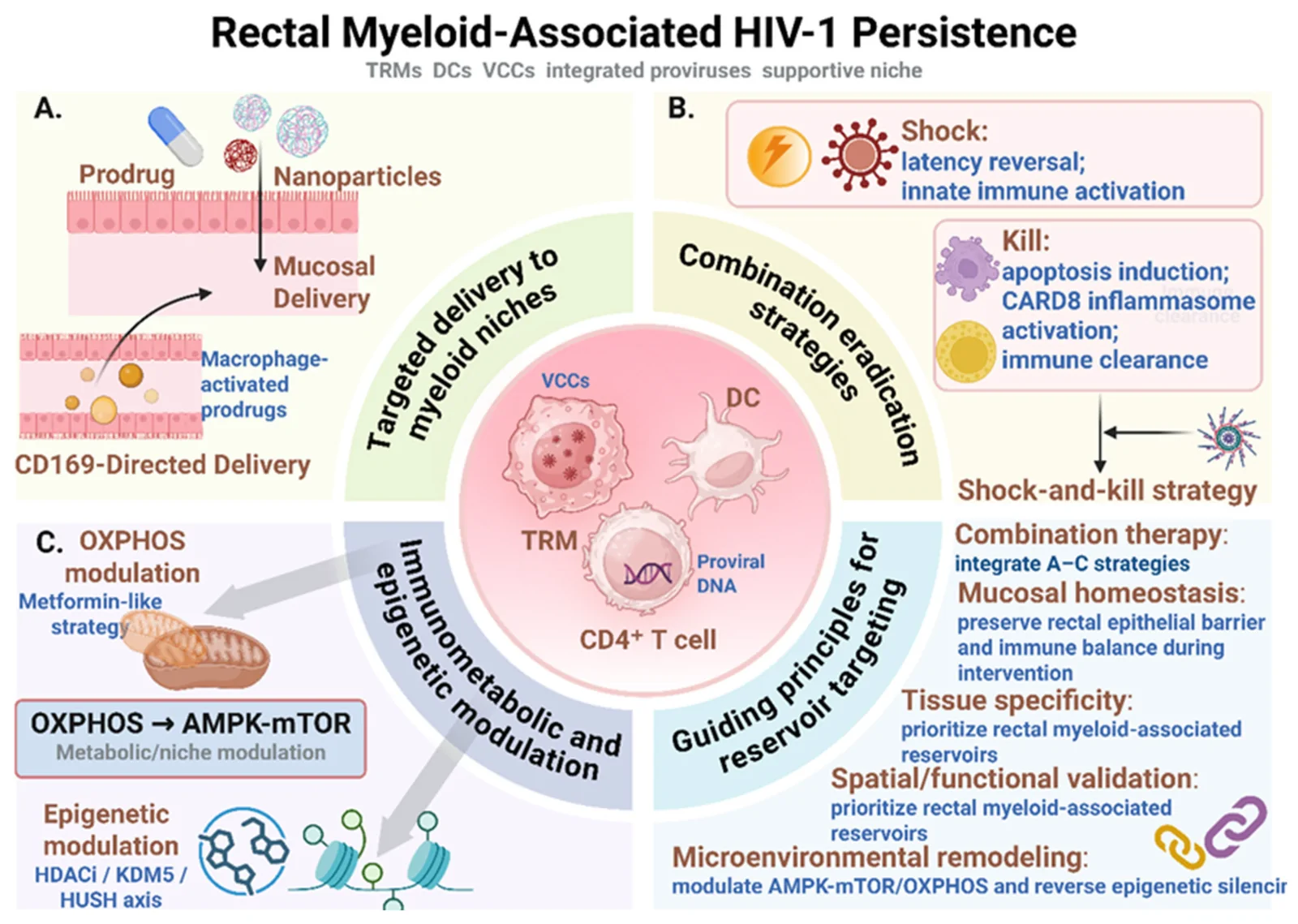
A translational roadmap for targeting rectal myeloid-associated HIV-1 persistence. Future therapeutic strategies targeting rectal myeloid-associated HIV-1 persistence may require complementary approaches rather than single-agent interventions. (**A**) Targeted drug delivery. This includes nanoparticle-based or liposome-based formulations, myeloid- or macrophage-targeted prodrugs, CD169-directed delivery systems, and approaches that improve mucosal retention and tissue penetration. (**B**) Combination eradication approaches. These approaches may integrate latency reversal or innate immune activation with pro-apoptotic sensitization, CARD8 inflammasome activation, survival-pathway inhibition, and immune-mediated clearance. (**C**) Immunometabolic and epigenetic modulation. Additional approaches may focus on HDAC inhibition, reversal of epigenetic silencing, metformin-associated metabolic reprogramming, OXPHOS targeting, AMPK–mTOR modulation, and microenvironmental remodeling. These approaches should be guided by tissue specificity, preservation of mucosal immune homeostasis, and spatial or functional validation. Created in BioRender. Zhang, H. (2026) https://BioRender.com/id669os.

Together, these considerations support a translational roadmap in which rectal myeloid-associated HIV-1 persistence is targeted through complementary rather than single-agent interventions. As summarized in Figure 3, future strategies should integrate tissue-targeted drug delivery, combination eradication approaches that couple latency reversal or innate immune activation with survival-pathway inhibition or immune-mediated clearance, and immunometabolic or epigenetic modulation to reshape the cellular and tissue states that stabilize persistence. These interventions should be guided by tissue specificity, preservation of mucosal immune homeostasis, and spatial or functional validation to distinguish reservoir-bearing cells from reservoir-supportive niches.

## 5. Conclusions and Perspectives

Achieving a functional HIV-1 cure increasingly requires a deeper understanding of viral persistence within complex tissue ecosystems. The rectal mucosa is more than a passive site of viral storage; it is a dynamic immune and metabolic niche in which epithelial barrier signals, microbial products, inflammatory cues, lymphovascular structures, and tissue-resident immune cells interact to influence HIV-1 persistence. Within this environment, non-CD4^+^ cell populations, particularly tissue-resident macrophages and dendritic cells, may contribute to persistence through reservoir-bearing states, viral transfer functions or reservoir-supportive components of the local niche.

Macrophages may support HIV-1 persistence through their long cellular lifespan, resistance to apoptosis, low-turnover tissue residency, metabolic adaptation, epigenetic regulation, and sequestration of virions within virus-containing compartments. Dendritic cells, in contrast, are best understood primarily as immune sentinels and viral transfer–competent cells that can capture HIV-1, facilitate trans-infection, regulate interferon and T-cell responses, and, in selected anatomical contexts, exhibit inducible proviral persistence. These mechanisms are distinct from classical CD4^+^ T-cell latency and therefore require different conceptual and experimental frameworks.

Despite growing interest in myeloid-associated HIV-1 persistence, major gaps remain in detection, quantification, and therapeutic targeting. Conventional blood-based assays and bulk tissue measurements cannot fully resolve cellular origin, proviral intactness, inducibility, or spatial localization. Future progress will require integrated approaches combining intact proviral assays, functional inducibility measurements, single-cell multi-omics, multiplex imaging, and spatial transcriptomics. Therapeutically, future strategies should move beyond a purely T-cell-centered model and incorporate tissue-targeted delivery, myeloid-specific latency modulation, immune clearance, and microenvironmental remodeling.

A comprehensive HIV-1 cure strategy should therefore address both infected cells and the tissue niches that support their persistence. By defining how rectal macrophages, dendritic cells, microbial metabolites, epithelial barrier disruption, and local immune regulation interact, future studies may identify more precise and durable interventions. This niche-informed perspective provides a framework for developing HIV-1 cure strategies that integrate antiviral activity with spatial resolution, cellular specificity, and immunological targeting.

## Figures and Tables

**Table 1:** Operational definitions of HIV-1 persistence in myeloid cells.

Concept	Operational Definition	Evidence Supporting Classification	Macrophages	Dendritic Cells
Viral capture/retention	Binding or storage of HIV-1 particles without proven infection.	Cell-associated virions or viral proteins, without confirmed integration.	Virions may be retained in intracellular compartments.	Efficient virion capture through lectin receptors.
Trans-infection	Transfer of captured virions to susceptible CD4^+^ T cells.	Receptor-dependent virion transfer or infectious synapse formation.	Can mediate cell-to-cell viral transfer.	Major mechanism of mucosal HIV-1 dissemination.
Productive infection	Active viral replication within a myeloid cell.	Viral RNA/protein production and, ideally, infectious virus release.	Supported in selected macrophage populations.	Generally inefficient, but possible in selected contexts.
Integrated provirus	HIV-1 DNA integrated into the myeloid-cell genome.	Integration-site analysis, Alu-PCR, or full-length proviral sequencing.	Reported in tissue-associated macrophages.	Reported mainly in selected myeloid DCs or lymphoid tissues.
Inducible latent reservoir	Integrated provirus that can be reactivated after stimulation.	Inducible viral RNA/protein or replication-competent virus.	Possible in selected tissue macrophages.	Possible, but evidence in rectal mucosal DCs remains limited.
Reservoir-supportive niche	A cell or tissue state that promotes persistence without necessarily harboring integrated provirus.	Viral transfer, immune suppression, impaired clearance, or spatial protection.	Supports persistence through longevity, VCCs, and immune regulation.	Supports persistence through capture, trans-infection, and immune modulation.

## Data Availability

No new data were created or analyzed in this study. Data sharing is not applicable to this article.

## Abbreviations

The following abbreviations are used in this manuscript:

ART	Antiretroviral therapy
cDC1	conventional DC1
cDC2	conventional DC2
DC	Dendritic cell
GALT	Gut-associated lymphoid tissue
moDC	Monocyte-derived DC
LRA	Latency-reversing agent
OXPHOS	Oxidative phosphorylation
pDC	Plasmacytoid DC
QVOA	Quantitative viral outgrowth assay
VCC	Virus-containing compartment
